# A systematic review of behaviour change techniques used in interventions to increase physical activity among breast cancer survivors

**DOI:** 10.1007/s12282-021-01323-z

**Published:** 2022-01-06

**Authors:** Verity Hailey, Antonio Rojas-Garcia, Angelos P. Kassianos

**Affiliations:** 1 Institute of Epidemiology and Healthcare, University College London, London, UK; 2 Department of Applied Health Research, University College London, 1-19 Torrington Place, London, WC1E 7HB UK

**Keywords:** Behaviour change techniques, Breast cancer, Exercise, Physical activity, Survivorship

## Abstract

**Background:**

Despite evidence that physical activity (PA) can help reduce recurrence and mortality, many breast cancer survivors are less active than recommended levels. The aim of this systematic review is to advance our understanding of which behaviour change techniques (BCTs) have been used in interventions promoting breast cancer survivors’ PA and to evaluate their potential to increase PA.

**Methods:**

A systematic search was conducted in five databases (Medline; PsycInfo; Embase; CINAHL and Scopus) for studies published between 2005 and 2019. Following a rigorous screening process, 27 studies were retained. These were reviewed and analysed for quality, coded for BCTs (*k* = 0.65) and interventions categorised according to their potential to increase PA using an established methodology.

**Results:**

The majority of studies were moderate quality (64%). Demonstration on how to perform the behaviour was the most commonly used BCT (*n* = 23). Adding objects to the environment, (pedometer or accelerometer) was the BCT with the highest potential to increase PA. This was followed by, goal setting and self-monitoring of behaviour. A theory-based approach to evaluation was used in only 59% (*n* = 16) of the studies.

**Conclusions:**

The results of this review inform which BCTs have the potential to increase PA for breast cancer survivors and inform intervention development. Future research, is encouraged to properly report intervention procedures around dose and frequency of intervention components to allow for review and replication.

**Supplementary Information:**

The online version contains supplementary material available at 10.1007/s12282-021-01323-z.

## Background

Cancer is the leading cause of death worldwide. In developed countries like the UK for example, breast cancer is the most common form of cancer diagnosed in women with an incidence of 169.8 per 100,000. Although incidence is rising, breast cancer mortality rates are falling, resulting in a growing number of breast cancer survivors [1]. Advances in treatment have improved survival rates but the disease and treatments can have long-term impact and side effects on women’s physical and psychological health [2]. General guidance for breast cancer survivors includes maintaining a healthy weight, limiting consumption of high-calorie food, reducing alcohol, and engaging in physical activity (PA) [3, 4]. Current evidence suggests that PA is safe and cost-effective resulting in health benefits like improved physical functioning and quality of life and reduced fatigue and risk of cancer recurrence [5]. In addition, PA can mitigate the side effects of breast cancer diagnosis and therapy including depression, decreased muscular strength, decreased aerobic capacity, and weight gain [6].

There is also evidence that PA is associated with a reduction in breast cancer survivors’ cancer recurrence by 24%, cancer-related mortality by 34% and all-cause mortality by 41% [7]. The World Health Organisation (WHO) recommends that women aged 18–64 should undertake at least 150 min of moderate-intensity PA or 75 min of vigorous PA and two sessions of resistance exercise each week [8]. Women who meet these guidelines also have improved psychosocial outcomes, specifically less fatigue and better quality of life when they are active over sustained periods of time [9].

Despite the evidence, many breast cancer survivors do not adhere to recommendations and their levels of PA are often worse than before diagnosis [5, 10]. Studies have looked at various ways of increasing rates of PA in this population, however, evidence suggests that although PA levels may increase, they subsequently fall back to baseline levels after the intervention [11, 12]. Therefore, there is a need to further understand which aspects or components of interventions are effective to increase PA.

Behaviour change interventions involve a number of interacting components making them complex, and therefore difficult to identify which part of the intervention is both active and effective. Recently a classification system on behaviour change techniques (BCTs) was developed with international consensus that aims to identify the active components of interventions [13, 14]. The BCTs constitute components that are used in behavioural interventions to optimise behaviour change and has been shown to improve intervention specifications in published reports and enable replication, evidence synthesis and implementation of PA programmes [15].

There is general agreement that a range of interventions can be effective in promoting modest increase in PA [2, 16–18]. There is a systematic review of PA interventions for prostate cancer survivors [19] but breast cancer survivors have a different demographic profile in that they are mostly female, younger and generally diagnosed at an earlier stage [2]. A previous meta-analysis [20] of behaviour change interventions targeting PA among breast cancer survivors concentrated on the setting, frequency, level of supervision and mode of intervention whilst the components of the interventions were not identified or categorised for effectiveness. It was noted that further investigation into the components of the intervention and the type of behavioural theory used will be useful. A review looking specifically at BCTs and their effectiveness in breast cancer is needed to inform on the type of intervention components available, future intervention development for PA change and maintenance [21].

The aim of this systematic review is to advance our understanding of which BCTs have been used specifically with breast cancer survivors and to evaluate their potential to increase PA levels. This is done by systematically reviewing the literature and exploring the effectiveness of BCTs by calculating a promise ratio [20] according to their potential to increase PA among breast cancer survivors.

## Materials and methods

The review was undertaken following the Preferred Reporting Items for Systematic Reviews and Meta-Analysis (PRISMA) guidelines [22] and pre-registered with PROSPERO (reference: CRD42019161188).

### Search strategy

Five databases were searched: Medline; PsycInfo; Embase; CINAHL and Scopus. Timeframe was limited to the last 15 years (2005–2019) due to multiple systematic reviews of PA behaviour change in cancer survivors reporting no studies prior to 2005 [2, 16–18]. The search terms were developed in Medline and adapted for use in the other databases and were developed from key concepts including: breast cancer, breast neoplasm, cancer survivor, exercise, physical activity, motor activity and randomised controlled trials. A detailed listing of search terms is available in Appendix 1.

### Study selection

Study selection was undertaken in two stages, (a) title and abstract screening for relevance and suitability and then (b) full text screening. The inclusion criteria for the studies were structured according to the PICO model: the population (women diagnosed with breast cancer who had completed treatment, except hormonal therapy within the past 5 years); the intervention (supervised or unsupervised PA, exercise, use and type of theory used,); the comparison (usual care or ‘waiting list’ control); and the outcome (a direct measure of PA).

Included studies could be randomized controlled trials (RCTs) or quasi-experimental studies with a comparison group. Cross sectional or qualitative studies were excluded. All eligible studies available in English language and published in peer reviewed journals were eligible to be included. Studies were excluded when the studies included other types of cancer where the results were reported as a mixed cancer cohort and when the intervention was reported in insufficient detail to code the BCTs. A second reviewer screened 10% of the results at both stages and discrepancies were resolved by discussion with a third reviewer.

### Data extraction

Data were extracted in four categories; study (author and year), population (sample size, age, ethnicity, education level), intervention (type, duration and theory) and outcomes (PA measurement). Summaries of the data were extracted in a spreadsheet developed for this review. A second author also assessed 10% of the extracted data independently.

#### Behaviour change technique’s coding

The BCT Taxonomy Version 1 [13, 23] was used to code the BCTs. The BCT taxonomy is acknowledged as the standard for identifying and coding interventions for health behaviour change. Online training was undertaken by coders to ensure understanding and consistency of coding. A second coder did 10% of included papers, and there was initial moderate inter-rater reliability [24] between coders (*k* = 0.65). This was attributed to lack of clarity in interventions’ description and in subjectivity entailed in systematic review quality assurance processes that require binary decisions (include/exclude) [25]. To resolve this, a series of consensus meetings were held between coders to reach a consensus on discrepancies. During these meetings, BCTs where coders were uncertain whether they were present were excluded from further analyses.

#### Risk of bias

The risk of bias of the studies was assessed using the Cochrane Collaboration’s Risk of Bias Tool [26]. The tool consists of questions to help assess the quality concerning six different sources of bias; selection bias, performance bias, detection bias, attrition bias, reporting bias and other bias. The risk of bias was coded for each study description, this was summed and described as ‘high’, ‘some concerns’ and ‘low’. Once assessment was complete, 10% was checked by a second assessor. Any discrepancies were resolved in a consensus meeting.

### Narrative synthesis and BCT efficacy

To investigate the potential contribution of BCTs used in an intervention on participants’ PA levels, a 'promise ratio’ developed by Gardner et al. [20] and previously used by Hallward et al. [19] and Grimmett et al. [2] was used to produce a narrative analysis of the quantitative results. Each study was allocated into one of three categories according to their potential to increase PA. Interventions were categorised as ‘very promising’, ‘quite promising’ and ‘non-promising’ based on whether within-group (intervention) and between-group (intervention and control) analyses demonstrated statistically significant increases in PA outcomes: (a) ‘very promising’ were those reporting statistically significant between-group differences in PA (intervention vs control); (b) ‘quite promising’ were those reporting within-group differences in the intervention group at post-intervention follow up, or a greater increase than the change observed in the control group; (c) ‘non promising’ were those reporting no statistically significant increase in PA within the intervention group (before and after) nor differences relative to the control group.

In addition, and to evaluate the contribution of BCTs used in an intervention, a ‘promise ratio’ was also calculated for each BCT. A BCT was considered promising if it was used in at least twice as many promising interventions as non-promising interventions (promise ratio > 2). If a BCT only appeared in promising interventions, the number of interventions in which it was used was reported as a score rather than a ratio. To prevent making conclusions with insufficient evidence, a promise ratio was not calculated for BCTs that only appeared in non-promising interventions or only appeared once. The higher the ratio the more promising an intervention. The highest possible score was 24, where a BCT was only found in promising or quite promising interventions. The lowest ratio would be 0.33, where a BCT was found once in a promising intervention and in all three of the non-promising interventions.

## Results

**Fig. 1:**
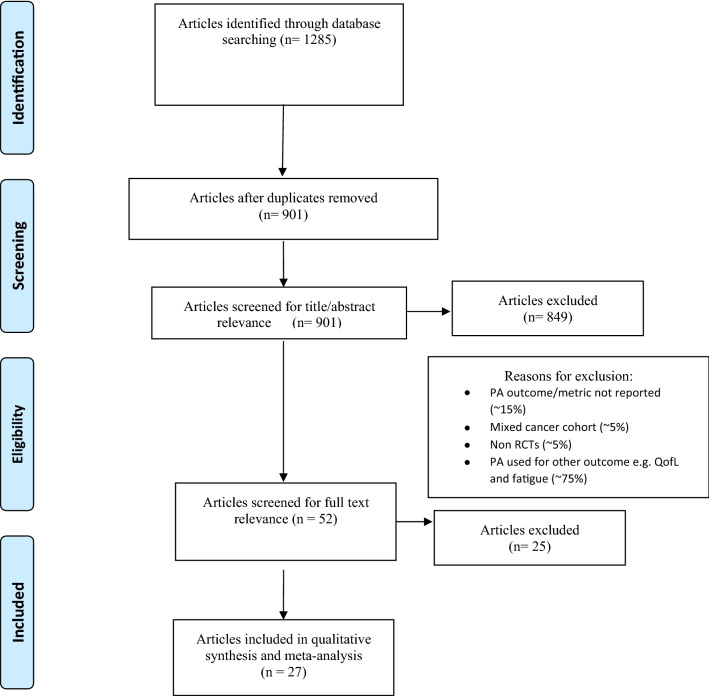
Flowchart for study selection (PRISMA-based)

Following the search, 1285 titles were identified, and removal of duplicates reduced this to 901 (Fig. 1). Screening of abstracts and titles produced 52 full-text articles to be reviewed. Twenty-seven studies were retained, data extracted and coded for the review. The majority (75%) of excluded studies, were excluded because they did not include a PA measure as an outcome.

### Study descriptions

A summary of participants’ and intervention characteristics is provided in Table 1 and more information provided in Supplementary Material. Of the 27 studies included in the review the majority of studies (59%) were conducted in the USA (*n* = 16) followed by Europe (*n* = 6), Australia (*n* = 2), Canada (*n* = 2) and South Korea (*n* = 1). All studies were RCTs except Leclerc et al. [27], which was quasi-experimental. Mean age of participants ranged from 45.6 (SD = 6.3) years to 61.6 (SD = 6.4) years. The total number of participants across all 27 studies was 3,656. The most prevalent ethnicity was ‘white’, with two studies exclusively looking at black women [28, 29] and one study having a majority Hispanic ethnicity (78.6%) [30]. Participants were of various stages of cancer at diagnosis with the majority (*n* = 2016, 58%) reported an early cancer diagnosis at stage I or stage II of the disease. The majority received surgery plus adjuvant therapy (radiotherapy or chemotherapy). The participants were mostly highly educated.

**Table 1:** Details of the study, population, intervention and outcome

Study		Population				Intervention Setting	Intervention		Outcome		RoB	
First author (year), country	Research design	Sample size	Mean age years	Main ethnicity	Main education level		Duration	Theory used	Measurement	Potential to increase PA	Cochrane overall bias	
Basen-Engquist (2006) USA	RCT	*N* = 60	55.05 (SD ± 11.4)	White (56.6%)	Some college (33%)	Delivered by unspecified experts with emphasis on self-regulation	26 weeks	TTM & TPB	MET-hours/week	Quite	High	
Baumann (2017) Germany	Quasi RCT	*N* = 194	56 (SD ± 9)	Not recorded	Not recorded	Delivered by physiotherapists with home-based programme	104 weeks	No	MET-min/week	Quite	High	
McNeil (2019) Canada	RCT	*N* = 45	58.6 (SD ± 9)	White (80%)	≥ Secondary school (82.2%)	Delivered at home using activity tracker	12 weeks 24 weeks follow up	No	MVPA min/day	Very @12 weeks Quite @24 weeks	Some concerns	
Daley (2007) UK	RCT	*N* = 108	51.1 (SD ± 8.7)	White (100%)	Finished high school (43.5%)	Delivered by exercise specialist at University centre	8 weeks	No	8-min walk test	Quite	Some concerns	
De Luca (2016) Italy	pilot RCT	*N* = 20	45.6 (SD ± 6.3)	Not recorded	Not recorded	Delivered by expert using aerobic and training exercises	24 weeks	No	VO2 max	Quite	Low	
Greenlee (2013) USA	RCT	*N* = 42	51 (SD ± 8.8)	Hispanic (78.6%)	≤ High school (30%)	Delivered at a fitness centre	26 weeks 26 weeks follow up	No		Very @26 weeks Non @ 52 weeks	Some concerns	
Harrigan (2016) USA	RCT	*N* = 100	59 (SD ± 7.5)	White (91%)	University (37%)	Delivered using an adapted diabetes prevention programme by telephone and in person	26 weeks	SCT	MVPA min/week	Very	Some concerns	
Hatchett (2013) USA	RCT	*N* = 74	Not recorded	White (94.6%)	Graduate degree (26%)	Delivered online using emails and an e-counsellor	12 weeks	SCT	MPA min/week	Quite	Some concerns	
Hirschey (2018) USA	RCT	*N* = 60	59 (SD ± 11)	White (74%)	Not recorded	Delivered by mail instructions	12 weeks	Not specified	MVPA min/week	Quite	High	
Irwin (2008) USA	RCT	*N* = 75	55.8 (SD ± 9.5)	White (84%)	University (50.5%)	Delivered by exercise physiologists face to face	26 weeks	TTM	MVPA min/week	Very	Some concerns	
Kim (2011) South Korea	RCT	*N* = 55	45.9 (SD ± 8.6)	Not recorded	≤ High school (52%)	Delivered using telephone counselling	12 weeks	TTM	MET-hours/week	Quite	Low	
Lahart (2016) UK	RCT	*N* = 80	53.6 (SD ± 9.4)	White (97%)	University (40%)	Delivered by researchers on site and then using virtual methods	26 weeks	No	MET-min/week	Very	Some concerns	
Lahart (2018) UK	RCT	*N* = 32	52.3 (SD ± 9.6)	White (100%)	Average 16.9 years	Delivered by researchers on site and then using virtual methods	26 weeks	No	*V*O_2_ max	Quite	Some concerns	
Leclerc (2018) Belgium	Quasi experimental	*N* = 209	53.3 (SD ± 9.3)	Not recorded	Not recorded	Delivered by physiotherapists in groups followed by psychoeducation	11 weeks	No	MET-hours/week	Non	High	
Lynch (2019) Australia	RCT	*N* = 83	61.6 (SD ± 6.4)	Not recorded	University (45.8%)	Delivered using a behavioural feedback and goal setting session and telephone behavioural counselling	12 weeks	No	MVPA min/week	Very	Some concerns	
Matthews (2007) USA	RCT	*N* = 36	54.1 (SD ± 10.6)	White (82%)	Not recorded	Delivered at home including in person counselling visit and telephone consultation	12 weeks	SCT	MPA min/day	Quite	Some concerns	
Park (2016) USA	RCT	*N* = 173	56.4 (SD ± 10.9)	White (94.7%)	University (24%)	Delivered by mail	16 weeks	No	MVPA min/week	Quite	Some concerns	
Pinto (2005) USA	RCT	*N* = 85	53.14 (SD ± 9.1)	White (96.4%)	Some college (28%)	Delivered at home including telephone consultation	12 weeks 12 weeks follow up	TTM	MPA min/week	Very	High	
Pinto (2013) USA	RCT	*N* = 192	60 (SD ± 9.9)	White (93.7%)	Some college (27%)	Delivered by healthcare professional recommendation followed by telephone consultations	12 weeks	TTM & SCT	MPA min/week	Very @12wks Quite @52 weeks	Some concerns	
Pinto (2015) USA	RCT	*N* = 76	55.6 (SD ± 9.6)	White (98.7%)	Some college (89%)	Delivered by other volunteer breast cancer survivors (peers)	12 weeks	TTM & SCT	MVPA min/week	Very	Low	
Rogers (2009) USA	RCT	*N* = 41	53 (SD ± 9)	White (93%)	Average 15 years	Delivered by experts using a behaviour change programme	12 weeks	SCT	MPA min/day	Quite	Some concerns	
Rogers (2014) USA	pilot RCT	*N* = 46	56.2 (SD ± 7.7)	White (95.5%)	Average 14 years	Delivered by experts including aerobic exercises	12 weeks	Biobehavioral models of fatigue	MVPA min/week	Very	Some concerns	
Rogers (2015) USA	RCT	*N* = 222	54.4 (SD ± 8.5)	White (83.8%)	Average 15.5 years	Delivered by experts including aerobic and home exercises	12 weeks	SCT	MVPA min/week	Very @12wks Quite @24 weeks	Low	
Saarto (2012) Finland	RCT	*N* = 573	52.3 (range 36–68)	Not recorded	Average 14 years	Delivered using physical exercise training	52 weeks	No	MET-hours/week	Non	Some concerns	
Sheppard (2016) USA	RCT	*N* = 22	Not recorded	Black (100%)	Not recorded	Delivered using individualised sessions	12 weeks	TPB	MET-min/week	Non	Some concerns	
Short (2015) Australia	RCT	*N* = 330	56 (range 33–82)	Not recorded	University (44%)	Delivered by mail using 3 methods (computer-tailored newsletters, pamphlets and cancer specific physical activity booklet)	16 weeks	SCT & TPB	MVPA min/week	Quite	Low	
Stolley (2017) USA	RCT	*N* = 246	57.5 (SD ± 10.1)	Black (100%)	Some college (37.9%)	Delivered using a cognitive-behavioural weight loss programme	26 weeks	SEM	MPA min/week	Quite	Some concerns	
Vallance (2007) Canada	RCT	*N* = 377	58 (range 30–90)	Not recorded	Not recorded	Delivered by intervention materials including guidance book	12 weeks	TPB	MVPA min/week	Very	Some concerns	

Biobehavioral, Biobehavioral models of fatigue (29); MET, metabolic equivalent; MPA, moderate physical activity; MVPA, moderate vigorous physical activity; PA, physical activity; RCT, randomised controlled trial; RoB, risk of bias assessment; SCT, social cognitive theory (30); SEM, socio ecological model (31); TPB, theory of planned behaviour (32); TTM, transtheoretical Model (33)

### Risk of bias

**Fig. 2:**
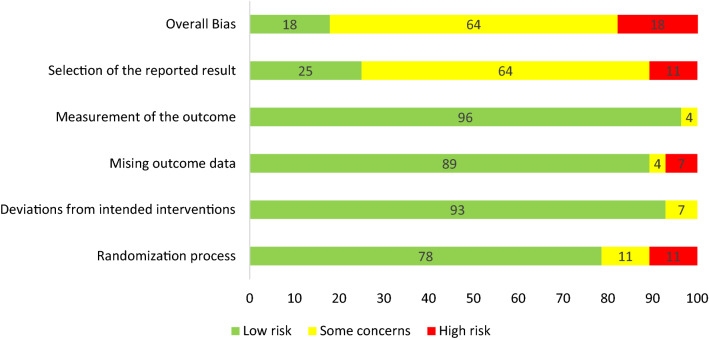
Quality assessment results presented as percentage across all studies (*n* = 27)

Overall, five (18%) of the 27 studies were rated as strong, 17 (64%) as moderate and five (18%) as low quality (Fig. 2). Randomisation and selection of the reported results were areas that potentially introduced bias. Sample size calculations were conducted in 56% of studies. Attrition of less than 20% for studies conducted for less than six months was achieved in 68% of studies. All studies specified inclusion criteria and the randomization and allocation methods were reported. The five low quality papers were not removed from the analysis to enrich methodological considerations but we based recommendations based on high-quality studies only.

### Type and classification of interventions

Study interventions included aerobic training like walking (*n* = 21, 78%) or a combination of aerobic and resistance training (*n* = 6, 22%). They were conducted in a gym/community setting (*n* = 5, 19%), at home (*n* = 15, 56%) or both (*n* = 7, 26%). Interventions lasted from eight to 104 weeks. All interventions encouraged weekly PA, with the aim to reach national recommended levels. The studies were classified into three promise categories, based on the potential of the intervention to increase PA behaviour (see Supplementary Material); ‘very promising’ (*n* = 11), ‘quite promising’ (*n* = 13) or ‘non-promising’ (*n* = 3).

In the included studies, PA was assessed using self-report as well as more objective measures such as pedometers and accelerometers which were generally more accurate [31]. The majority of studies reported MVPA min/week, (*n* = 17, 63%), followed by MET-hours/week (*n* = 7, 26%). Of the 11 studies that were ‘very promising’, the majority reported MVPA mins/week (*n* = 10) with only one study reporting MET-hours/week. For the thirteen ‘quite promising’ studies 10 reported objective measures: 8-min walk test (*n* = 1), VO_2_ max test (*n* = 2), MVPA min/week (*n* = 7), with the remaining studies reporting MET-hours/week (*n* = 3). The three ‘non-promising’ studies all reported MET-hours/week.

A theory-based approach to evaluation was used in 59% (*n* = 16) of the studies. Of the twenty-four promising studies, fifteen studies (62%) were theoretically-based. 11 studies (46%) were based on a single theory, and four studies had used multiple theories (16%). Of the three non-promising studies, two (67%) were not theoretically-based.

Five different approaches were used; social cognitive theory (SCT) [32, 33] (*n* = 8), transtheoretical model of change (TTM) [34, 35] (*n* = 6), theory of planned behaviour (TPB) [36] (*n* = 3), biobehavioural model of fatigue [37] (*n* = 1), social ecological model (SEM) [38] (*n* = 1), whilst in one study the approach was not specified (*n* = 1). Four studies used multiple theories. The TTM and SCT were used together twice and both studies were ‘very promising’. The combination of TTM with TPB, and TPB with SCT, were also ‘quite promising’.

### Behaviour change techniques (BCTs)

Out of the possible 93 BCTs, 24 were used at least once in an intervention in the included studies with an average of six BCTs per study (range 3–9). The median number of BCTs used was indifferent between ‘very promising’ (*n* = 6), ‘quite promising’ (*n* = 6) and ‘non-promising’ interventions (*n* = 7).

No BCT was used in all interventions. The most frequently reported BCTs were; ‘instructions on how to perform the behaviour’ (*n* = 23, 85%), ‘goal setting (behaviour)’ (*n* = 22, 81%), ‘adding objects to the environment’ (*n* = 13, 48%), ‘self-monitoring of behaviour’ (*n* = 11, 41%) and ‘demonstration of the behaviour’ (*n* = 10, 37%) (Supplementary Material).

Twenty BCTs were found to be promising, for which 13 could have promise ratio calculated. Table 2 outlines the BCTs’ promise ratio. One BCT (‘restructuring the physical environment’) appeared only in a ‘very promising’ study [30]. There were five BCTs which appeared only in ‘quite promising’ interventions; ‘action planning’, ‘monitoring of outcomes of behaviour by other without feedback’, ‘biofeedback’, ‘social comparison’, ‘social reward’. There were five BCTs which appeared in both, ‘very’ or ‘quite promising’ interventions; ‘review behaviour goals’, ‘monitoring of behaviour by other without feedback’, ‘feedback on behaviour’, ‘self-monitoring of outcome of behaviour’ and ‘prompts/cues’. No BCT appeared only in non-promising interventions.

**Table 2:** BCT implemented per type of intervention and promise ratio

	Types of interventions					
	Very promising (*n* = 11)	Quite promising (*n* = 13)	Non-promising (*n* = 3)	All (*n* = 27)		Promise ratio^†^ OR *Number*^‡^
**1.1 Goal setting (behaviour)**	11	9	2	22		10
**1.2 Problem solving**	5	3	1	9		9
1.4 Action planning	0	1	0	1		1
1.5 Review behaviour goals	1	1	0	2		*2*
**2.1 monitoring of behaviour by others without feedback**	2	1	0	3		*3*
**2.2 Feedback on behaviour**	5	2	0	7		*7*
**2.3 Self-monitoring of behaviour**	5	5	1	11		11
**2.4 Self-monitoring of outcomes of behaviour**	4	3	0	7		*7*
**2.5 Monitoring of outcomes of behaviour without feedback**	0	3	0	3		*3*
2.6 Biofeedback	0	2	0	2		*2*
**3.1 Social support (unspecified)**	1	4	2	7		2.5
**3.2 Social support (practical)**	3	4	1	8		8
**3.3 Social support (emotional)**	4	2	1	7		7
**4.1 Instruction on how to perform the behaviour**	9	11	3	23		6.66
**5.1 Information on health consequences**	2	3	1	6		6
**6.1 Demonstration of the behaviour**	3	4	3	10		2.33
6.2 Social comparison	0	1	0	1		1
7.1 Prompts/cues	1	1	0	2		*2*
**8.1 Behavioural practice/rehearsal**	1	2	1	4		4
**9.1 Credible source**	2	2	1	5		5
**10.1 Material incentive (behaviour)**	2	3	1	6		6
10.4 Social reward	0	2	0	2		*2*
12.1 Restructuring the physical environment	1	0	0	1		1
**12.5 Adding objects to the environment**	9	3	1	13		13

^†^Promise ratio denotes the number of very or quite-promising interventions in which a behaviour change technique occurred divided by the number of non-promising interventions in which it featured. Rows in bold denote BCTs associated with a promise rate > 2 or used in promising interventions in at least two interventions

^‡^If a BCT only appeared in promising interventions and in at least two intervention, the number of interventions in which it appeared is reported in italics

In Table 1, we have also specified the intervention settings. These were diverse in nature but some patterns were observed including the use of home-based (*n* = 5), virtual (*n* = 3), telephone (*n* = 6) and only one using peer-to-peer approaches (see Table 1). Interestingly, not all home-based interventions used self-monitoring approaches such as self-monitoring of outcomes of behaviour with some specifying this [39] and others not [40, 41]. Also, those using telephone consulting approaches have incorporated BCTs related to feedback on how the participants performed [39, 42–44].

## Discussion

Setting goals and measuring activity are found consistently effective in increasing PA [12, 45], suggesting that these studies were conducted in line with current research. Social prescribing programs can help empower patients through social, emotional and practical support leading to increased PA and improved psychosocial outcomes. The BCTs identified in this review are similar with those found in other systematic reviews looking at increasing PA in other types of cancer survivors [16–18, 20]. For example, ‘instructions on how to perform the behaviour’ and ‘self-monitoring’ were effective across studies of other cancer survivors. All but one study also incorporated ‘adding objects to the environment’, associated with the use of a pedometer or an accelerometer. These BCTs can inform future etiologic studies that are needed to identify sub-groups that can benefit most from the interventions as previously suggested [46]. For example, on how self-monitoring can help patients already motivated to being active as opposed to those introduced to exercise post-diagnosis.

Intervention classification was used to examine the potential of the interventions to increase PA. A previous meta-analysis [20] of behaviour change interventions targeting physical activity among breast cancer survivors had concentrated on components like setting, frequency, level of supervision, and mode of intervention, without identifying the behavioral components for effectiveness and raised this as a limitation which we were attempting to address with this review. Due to the small number of studies which were classified as ‘non-promising’, the calculated promise ratios were large compared to other studies which have used this classification system [2, 19, 20, 47]. No differences were identified in number of BCTs between promising and non-promising interventions. However, ‘adding objects to the environment’ (pedometer or accelerometer) had the highest promise ratio followed by ‘goal setting’ and ‘self-monitoring of behaviour’. ‘Demonstration on how to perform the behaviour’ was the most commonly used BCT (*n* = 23) and was used in all three of the ‘non-promising’ interventions. There were five high-quality studies in total, three of which used all the promising BCTs. ‘Goal setting behaviour’ appeared in all five of the high-quality studies, ‘adding objects to the environment’ in three and ‘self-monitoring’ in two.

### Theoretical Implications

The majority of studies classified as promising were not theoretically-based even if a previous meta-analysis showed that theory-based interventions significantly increase PA [48]. A possible explanation may be that many health behaviour theories, such as the ones used in the interventions included in this review, focus on initiating or predicting a behaviour and do not consider long-term maintenance of behaviour. Interventions need to support change involving both psychosocial and behaviour-shift for long-term behaviour change [10] which provides ongoing clinical benefit. Studies that are not theoretically-informed are often focused on identifying the effective intervention technique for specific behaviours or populations whereas theory-based interventions tend to emphasise individual capabilities and motivation [49].

A recent systematic review of behaviour change interventions used for increasing PA in breast cancer survivors suggested that extensive use of theoretical frameworks can impact intervention effectiveness [50]. We found that social cognitive theory (SCT) was the most frequently used theory. SCT is based on the idea of learning and doing and that learning occurs in a social context with an emphasis on social influence [51]. Key limitations of this theory are assumptions that a change in environment like adding a pedometer will automatically lead to changes in the behaviour ignoring emotions and motivations. Interestingly, this review identified ‘adding objects to the environment’ having the highest promise ratio suggesting that SCT can be used to inform interventions aiming to optimize PA and also that simple changes in the patients’ environment may lead to significant changes in PA. [10]. In general, the BCTs aligned with SCT have been found to have a positive effect on intention but not on actual behaviour change [52]. While this suggests that some aspects of SCT may work for increasing PA, the absence of emotional and motivational aspects within this theory need to be addressed for maximum benefit to occur two studies that used a combination of transtheoretical model (TTM) and SCT were both classified as very promising suggesting that this combination could be effective. Transtheoretical model assesses an individual's readiness to act on a new health behaviour, SCT supports the process of change through learning and acting in a social environment, providing a process to guide the individual to adopt new behaviours.

Future research could focus on building a suite of BCTs which link with each theory of change. When designing an intervention, the chosen theory would link with the suite of BCTs allowing for a cohesive and systematic approach to intervention development.

### Methodological considerations

The majority of the trials had an average duration of 12 weeks which is relatively short for a behaviour change to occur while other reviews have shown long-term follow up as beneficial [53]. Only four studies had a specified follow-up period, which demonstrates a drop-in effect. Furthermore, future studies will benefit from clarifying baseline PA levels and whether patients meet recommended local PA guidelines. This will inform on the clinical relevance of any changes in PA identified as a result of the interventions.

Across the majority of the 27 studies, high risk of bias was due to the randomisation process. As PA interventions are difficult to blind due to the nature of the intervention [54], very few studies were able to keep blinding participants, study staff or assessors. In seven studies [27, 28, 42–44, 55, 56] there was either no change, or a drop-in activity in the control arm during the study period. Baumann et al. [11] acknowledged the issue of contamination and conducted the trial across two separate sites, however this compromised randomisation. Waters et al. [48] noted that improvements in PA in the control group is not uncommon, and these changes can be of similar size to improvements seen in the intervention group due to contamination. There were some variations in transparency of reporting. High attrition was also seen in a number of studies. However, information on missing data and on how these were managed was not reported in most of the studies.

### Study limitations

Despite a comprehensive literature search, the majority of study participants were well educated, white women in their 50 s, diagnosed at an early stage of cancer, living in North America or Europe. Findings from these studies may not be generalizable to other settings. Also, some studies used self-reported PA measures which restricts this review to the potential of reporting bias. However, the majority (82%) of the studies used more objective measurement methods such as pedometers, to validate or confirm self-reported measures. In those studies where both self-reported and more objective measurement methods were used, we haven’t identified any pattern of under- or over- reporting of PA and thus we are confident that the BCTs’ impact on PA is robust.

A limitation when evaluating behaviour change interventions is that publications often do not have sufficient information to allow coding of BCTs. For this study, only studies with clear evidence of BCTs were coded. Moreover, another limitation of this review is the moderate agreement between coders when coding the interventions’ BCTs. However, the method used for identifying the BCTs was empirically developed and similar reviews found similar agreement rates of *k* = 0.68 [57].

The evidence on the effectiveness of BCTs should be considered with caution as the use of the promise ratio has limitations. Promise ratio assumes that the BCTs directly impact PA levels, however, PA may be affected by other factors as well. Also, promise rations do not account for mitigating declines as a result of the change caused by the intervention. For example, an intervention may aim to help women be active to mitigate functional decline with activity therefore resulting in a no within-group difference. Nonetheless, by systematically assessing the promise ratio of a number of BCTs, patterns of effectiveness can be particularly informative.

Finally, extracting BCTs can obscure the active contents of the interventions because they rely in general behaviour change principles rather than specific components. However, this approach allows for the content to be summarised at a macro rather than micro level which thus allows for the core concept rather than content of the intervention to be captured. It is advisable that researchers investigating the use of BCTs with the specific setting they incorporate and delivery methods they use for their interventions. For example, we need evidence on how best to incorporate feedback approaches when using telephone consultations during the intervention delivery, or self-monitoring approaches when delivering the interventions online and at home.

## Conclusions

In this review, there were a range of BCTs identified across the included studies demonstrating that multiple techniques were used and identified. Using self-regulation techniques in the context of health-related behaviour adherence, especially in terms of physical activity engagement, is only one aspect that is required for behaviour change such as behaviour-shift (monitored through self-regulation). However, for behaviour change to happen, motivation and opportunity to engage in behaviour are equally important and warrant consideration. In future research, authors should be encouraged to properly report intervention procedures such as dose and frequency of intervention components and how these were implemented. From this review; ‘instructions on how to perform the behaviour’, ‘goal setting’, ‘self-monitoring’ and adding a pedometer or accelerometer into the environment in combination appear to have a beneficial effect on PA. These BCTs encourage and support people to take responsibility for their own health which is important for any long-term condition. Incorporating these techniques in digital health interventions is also promising [58].

### Electronic supplementary material

Below is the link to the electronic supplementary material.

Supplementary file1 (DOCX 32 kb)

Supplementary file2 (DOCX 25 kb)

## Appendix 1

Appendix 1: Search terms/strategy used with Medline database
