# Supplementary material for: A systematic review of behaviour change techniques used in interventions to increase physical activity among breast cancer survivors
Source: Breast Cancer. 2022 Jan 6;29(2):193–208. doi: 10.1007/s12282-021-01323-z (PMC8885559; doi:10.1007/s12282-021-01323-z)
Supplement: Supplementary file 1 — Supplementary file1 (DOCX 32 kb) [file 12282_2021_1323_MOESM1_ESM.docx]

Table 2. Behaviour change techniques identified in each study

|  | **Very promising** | | | | | | | | | | |  |
| --- | --- | --- | --- | --- | --- | --- | --- | --- | --- | --- | --- | --- |
| BCT | Pinto (2005) | Vallance (2007) | Irwin (2008) | Greenlee (2013) | Rogers (2014) | Pinto (2015) | Rogers (2015) | Harrigan (2016) | Lahart (2016) | Brenner (2019) | Lynch (2019) | **Total** |
| 1.1 Goal setting (behaviour) | x | x | x | x | x | x | x | x | x | x | x | 11 |
| 1.2 Problem solving | x |  |  |  |  | x | x |  |  | x | x | 5 |
| 1.4 Action planning |  |  |  |  |  |  |  |  |  |  |  | 0 |
| 1.5 Review behaviour goals |  |  |  |  |  |  |  |  |  |  | x | 1 |
| 2.1 Monitoring of behaviour by others without feedback |  |  | x |  | x |  |  |  |  |  |  | 2 |
| 2.2 Feedback on behaviour | x |  |  | x |  | x |  |  |  | x | x | 5 |
| 2.3 Self-monitoring of behaviour | x |  |  |  |  | x |  | x | x |  | x | 5 |
| 2.4 Self-monitoring of outcomes of behaviour |  | x | x | x |  |  |  |  |  | x |  | 4 |
| 2.5 Monitoring of outcomes of behaviour without feedback |  |  |  |  |  |  |  |  |  |  |  | 0 |
| 2.6 Biofeedback |  |  |  |  |  |  |  |  |  |  |  | 0 |
| 3.1 Social support (unspecified) |  |  | x |  |  |  |  |  |  |  |  | 1 |
| 3.2 Social support (practical) | x |  |  |  |  |  |  |  |  | x | x | 3 |
| 3.3 Social support (emotional) |  |  |  |  |  | x | x | x | x |  |  | 4 |
| 4.1 Instruction on how to perform the behaviour | x | x | x | x | x | x | x | x | x |  |  | 9 |
| 5.1 Information on health consequences |  |  |  |  |  |  |  |  | x |  | x | 2 |
| 6.1 Demonstration of the behaviour |  |  |  |  | x |  | x |  | x |  |  | 3 |
| 6.2 Social comparison |  |  |  |  |  |  |  |  |  |  |  | 0 |
| 7.1 Prompts/cues | x |  |  |  |  |  |  |  |  |  |  | 1 |
| 8.1 Behavioural practice/rehearsal |  |  |  |  |  |  | x |  |  |  |  | 1 |
| 9.1 Credible source |  |  |  |  | x |  | x |  |  |  |  | 2 |
| 10.1 Material incentive (behaviour) |  |  |  |  | x |  |  |  |  | x |  | 2 |
| 10.4 Social reward |  |  |  |  |  |  |  |  |  |  |  | 0 |
| 12.1 Restructuring the physical environment |  |  |  | x |  |  |  |  |  |  |  | 1 |
| 12.5 Adding objects to the environment | x | x | x | x |  | x | x | x |  | x | x | 9 |
| **TOTAL BCTs** | 8 | 4 | 6 | 6 | 6 | 7 | 8 | 5 | 6 | 7 | 8 |  |

Table 2. Behaviour change techniques identified in each study (continued)

|  | **Quite promising** | | | | | | | | | | | | | **Not promising** | | |  |
| --- | --- | --- | --- | --- | --- | --- | --- | --- | --- | --- | --- | --- | --- | --- | --- | --- | --- |
|  | Basen (2006) | Daley (2007) | Matthews (2007) | Rogers (2009) | Kim (2011) | Hatchett (2013) | Short (2015) | De Luca (2016) | Park (2016) | Baumann (2017) | Stolley (2017) | Hirschey (2018) | Lahart (2018) | Saarto (2012) | Sheppard (2016) | Leclerc (2018) | **Total** |
| 1.1 Goal setting (behaviour) | x |  | x | x | x |  | X | x | x | x |  |  | x |  | x | x | 11 |
| 1.2 Problem solving |  |  | x |  |  | x |  |  |  | x |  |  |  |  | x |  | 4 |
| 1.4 Action planning |  |  |  |  |  | x |  |  |  |  |  |  |  |  |  |  | 1 |
| 1.5 Review behaviour goals |  |  |  |  |  |  |  |  | x |  |  |  |  |  |  |  | 1 |
| 2.1 Monitoring of behaviour by others without feedback |  |  |  |  |  |  | X |  |  |  |  |  |  |  |  |  | 1 |
| 2.2 Feedback on behaviour |  |  | x |  |  | x |  |  |  |  |  |  |  |  |  |  | 2 |
| 2.3 Self-monitoring of behaviour | x |  |  |  |  | x | X |  | x |  |  |  | x |  | x |  | 6 |
| 2.4 Self-monitoring of outcomes of behaviour |  |  | x | x | x |  |  |  |  |  |  |  |  |  |  |  | 3 |
| 2.5 Monitoring of outcomes of behaviour without feedback |  |  |  |  |  |  |  | x | x | x |  |  |  |  |  |  | 3 |
| 2.6 Biofeedback |  | x |  |  |  |  |  | x |  |  |  |  |  |  |  |  | 2 |
| 3.1 Social support (unspecified) |  |  | x |  |  |  |  |  |  | x | x |  | x | x | x |  | 6 |
| 3.2 Social support (practical) | x | x |  | x |  | x |  |  |  |  |  |  |  |  |  | x | 5 |
| 3.3 Social support (emotional) |  |  | x |  | x |  |  |  |  |  |  |  |  |  | x |  | 3 |
| 4.1 Instruction on how to perform the behaviour | x | x |  | x | x |  | X | x | x | x | x | x | x | x | x | x | 14 |
| 5.1 Information on health consequences | x |  |  |  |  |  |  |  |  |  | x | x |  |  |  | x | 4 |
| 6.1 Demonstration of the behaviour |  | x |  |  |  |  |  | x |  | x | x |  |  | x | x | x | 7 |
| 6.2 Social comparison |  |  |  |  |  |  |  |  |  |  | x |  |  |  |  |  | 1 |
| 7.1 Prompts/cues |  |  |  |  |  |  |  |  |  |  | x |  |  |  |  |  | 1 |
| 8.1 Behavioural practice/rehearsal | x |  |  |  |  |  |  | x |  |  |  |  |  |  |  | x | 3 |
| 9.1 Credible source |  |  |  |  |  |  |  | x |  |  |  | x |  |  |  | x | 3 |
| 10.1 Material incentive (behaviour) |  |  |  | x |  |  |  |  | x |  |  | x |  |  | x |  | 4 |
| 10.4 Social reward |  |  | x | x |  |  |  |  |  |  |  |  |  |  |  |  | 2 |
| 12.1 Restructuring the physical environment |  |  |  |  |  |  |  |  |  |  |  |  |  |  |  |  | 0 |
| 12.5 Adding objects to the environment | x |  |  |  | x |  |  |  |  |  |  | x |  |  | x |  | 4 |
| **TOTAL BCTs** | 7 | 4 | 7 | 6 | 5 | 5 | 4 | 7 | 6 | 6 | 6 | 5 | 4 | 3 | 9 | 7 |  |
