# Supplementary material for: A systematic review of behaviour change techniques used in interventions to increase physical activity among breast cancer survivors
Source: Breast Cancer. 2022 Jan 6;29(2):193–208. doi: 10.1007/s12282-021-01323-z (PMC8885559; doi:10.1007/s12282-021-01323-z)
Supplement: Supplementary file 2 — Supplementary file2 (DOCX 25 kb) [file 12282_2021_1323_MOESM2_ESM.docx]

Table 1. Details of the study, population, intervention and outcome

| **Study** | | **Population** | | | | **Intervention** | | | **Outcome** | | **RoB** |
| --- | --- | --- | --- | --- | --- | --- | --- | --- | --- | --- | --- |
| First author (year) | Country  (Design) | Sample size | Mean age years | Main ethnicity | Main education level | Description | Duration | Theory used | Measurement | Potential to increase PA | Cochrane overall bias |
| Pinto  (2005 )(1) | USA  (RCT) | N= 85 | 53.14  (SD ±9.1) | White  (96.4%) | Some college (28%) | In-person and telephone counselling. Home logs and pedometer to record & monitor PA. Goals set & feedback provided on progress.  Initial in-person session, weekly calls for 12 weeks, monthly calls for 12 weeks | 12 wks 12 wks follow up | TTM | MPA min/week |  | High |
| Basen-Engquist (2006) (2) | USA  (RCT) | N = 60 | 55.05  (SD ±11.4) | White  (56.6%) | Some college (33%) | Group meetings, weekly for 16 weeks, alternate weeks for 8 weeks. Home logs and pedometer to record and monitor PA.  Workshop and group exercise done in a community setting | 26 wks | TTM & TPB | MET-hrs/week | Quite | High |
| Daley  (2007) (3) | UK  (RCT) | N = 108 | 51.1  (SD ±8.7) | White  (100%) | Finished high school (43.5%) | In-person, individual exercise session with behaviour counselling.  3xweek for 8weeks | 8 wks | No | 8-min walk test | Quite | Some concerns |
| Matthews  (2007) (4) | USA  (RCT) | N = 36 | 54.1  (SD ±10.6) | White  (82%) | Not recorded | Home based walking intervention, in-person and telephone counselling. Home logs and pedometer to record & monitor PA. Goals set & feedback provided on progress.  Initial in-person session, 5 x telephone calls at set periods | 12 wks | SCT | MPA min/day | Quite | Some concerns |
| Vallance  (2007) (5) | Canada  (RCT) | N = 377 | 58  (range 30-90) | Not recorded | Not recorded | Exercise guide, pedometer and step calendar. Home based. No counselling support | 12 wks | TPB | MVPA min/week | Very | Some concerns |
| Irwin  (2008) (6) | USA  (RCT) | N = 75 | 55.8  (SD ±9.5) | White  (84%) | University (50.5%) | Individual behaviour counselling. Small group & home based training. Group training 3x week. Education material, exercise logs and pedometer/HR monitor provided. Self-monitoring and feedback provided. | 26 wks | TTM | MVPA min/week | Very | Some concerns |
| Rogers  (2009) (7) | USA  (RCT) | N = 41 | 53  (SD ±9) | White  (93%) | Average 15 years | Behaviour counselling and exercise sessions. Home based PA logged and converted to ‘miles’ for competition within group.  6 discussion group, 12 supervised exercise sessions, 3 counselling sessions | 12 wks | SCT | MPA min/day | Quite | Some concerns |
| Kim  (2011) (8) | Korea  (RCT) | N = 55 | 45.9  (SD ±8.6) | Not recorded | ≤high school (52%) | Telephone counselling, workbook and HR monitor. Homebased, exercise diary to record and monitor PA. Weekly calls for 12 weeks to set new goals. | 12 wks | TTM | MET-hrs/week | Quite | Low |
| Saarto  (2012) (9) | Finland  (RCT) | N = 573 | 52.3  (range 36-68) | Not recorded | Average 14 years | Behaviour counselling. Supervised group and home based exercise training. Weekly group session. | 52 wks | No | MET-hrs/week | Non | Some concerns |
| Greenlee  (2013) (10) | USA  (RCT) | N = 42 | 51  (SD ±8.8) | Hispanic  (78.6%) | ≤high school (30%) | Behaviour counselling.  3 supervised exercise session per week. | 26 wks 26 wks follow up | No |  | Very @26 wks  Non @ 52 wks | Some concerns |
| Hatchett  (2013) (11) | USA  (RCT) | N = 74 | Not recorded | White  (94.6%) | Graduate degree (26%) | Behaviour counselling via email. Weekly email for 5 weeks then every other week for 6 weeks. Access to an e-counsellor. Home based exercise. | 12 wks | SCT | MPA min/week | Quite | Some concerns |
| Pinto  (2013) (12) | USA  (RCT) | N = 192 | 60  (SD ±9.9) | White  (93.7%) | Some college (27%) | Behaviour counselling in person and then via phone. Initial session in person then 8 calls.  Home logs and pedometer to record and monitor PA. Review of PA and setting goals | 12 wks | TTM & SCT | MPA min/week | Very @12wks  Quite @52wks | Some concerns |
| Rogers  (2014) (13) | USA  (pilot RCT) | N = 46 | 56.2  (SD ±7.7) | White  (95.5%) | Average 14 years | Behaviour counselling in a group setting. Individual supervised exercise program, 3x week for 2 weeks, 2x week for 10 weeks. Six group meetings, every other week. Walking and strength training. | 12 wks | Biobehavioral models of fatigue | MVPA min/week | Very | Some concerns |
| Pinto  (2015) (14) | USA  (RCT) | N = 76 | 55.6  (SD ±9.6) | White  (98.7%) | Some college (89%) | Behaviour counselling via telephone. Weekly telephone calls. Home logs and pedometer and HR monitor to record & monitor PA. Feedback report x4. | 12 wks | TTM & SCT | MVPA min/week | Very | Low |
| Rogers  (2015) (15) | USA  (RCT) | N = 222 | 54.4  (SD ±8.5) | White  (83.8%) | Average 15.5 years | Behaviour counselling and exercise sessions. 12x session in first 6 weeks, then exercise home based. In person counselling every other week (3 session). 6x group session. Education notebook and HR monitor for self-monitoring. | 12 wks | SCT | MVPA min/week | Very @12wks  Quite @24wks | Low |
| Short  (2015) (16) | Australia  (RCT) | N = 330 | 56  (range 33-82) | Not recorded | University (44%) | Mail based intervention. Home based exercise, no counselling support. Three tailored newsletters six weeks apart. | 16 wks | SCT & TPB | MVPA min/week | Quite | Low |
| De Luca  (2016) (17) | Italy  (pilot RCT) | N = 20 | 45.6  (SD ±6.3) | Not recorded | Not recorded | In-person group exercise sessions. Twice weekly for 24 weeks. No behaviour counselling support. | 24 wks | No | VO2 max | Quite | Low |
| Harrigan  (2016) (18) | USA  (RCT) | N = 100 | 59  (SD ±7.5) | White  (91%) | University (37%) | Behaviour counselling, in-person or telephone. 11x over 26 weeks. Journal and pedometer to record & monitor PA. Home based | 26 wks | SCT | MVPA min/week | Very | Some concerns |
| Lahart  (2016) (19) | UK  (RCT) | N = 80 | 53.6  (SD ±9.4) | White  (97%) | University (40%) | Behaviour counselling, in-person & telephone. 1x face to face, 3x calls, 2x mailed leaflets. Home based. | 26 wks | No | MET-min/week | Very | Some concerns |
| Park  (2016) (20) | USA  (RCT) | N = 173 | 56.4  (SD ±10.9) | White  (94.7%) | University (24%) | Mail based program. Material sent every other week (8 total). Home logs completed and sent to progam. | 16 wks | No | MVPA min/week | Quite | Some concerns |
| Sheppard  (2016) (21) | USA  (RCT) | N = 22 | Not recorded | Black  (100%) | Not recorded | Behaviour counselling – group counselling and exercise every 2 weeks (6 session). Individually by phone, every 2 weeks (6 sessions)  Journal and pedometer to record & monitor PA, step goals | 12 wks | TPB | MET-min/week | Non | Some concerns |
| Baumann  (2017) (22) | Germany  (Quasi RCT) | N = 194 | 56  (SD ±9) | Not recorded | Not recorded | 3 week rehabilitation program, followed by home based, then further 1 week at 4 & 8months. 1x phone call after initial 3week stay. | 104 wks | No | MET-min/week | Quite | High |
| Stolley  (2017) (23) | USA  (RCT) | N = 246 | 57.5  (SD ±10.1) | Black  (100%) | Some college (37.9%) | Behaviour counselling in group setting with exercise. This occurred twice weekly. Twice weekly text support. Program binder with supportive material provided. | 26 wks | SEM | MPA min/week | Quite | Some concerns |
| Hirschey  (2018) (24) | USA  (RCT) | N = 60 | 59  (SD ±11) | White  (74%) | Not recorded | Paper booklet based intervention | 12 wks | Y  Not specified | MVPA min/week | Quite | High |
| Lahart  (2018) (25) | UK  (RCT) | N = 32 | 52.3  (SD ±9.6) | White  (100%) | Average 16.9 years | Behaviour counselling in-person x1 and by phone x3. Postcard prompts x2. Written information and DVD provided at start. | 26 wks | No | VO2 max | Quite | Some concerns |
| Leclerc  (2018) (26) | Belgium  (Quasi experimenatl) | N = 209 | 53.3  (SD ±9.3) | Not recorded | Not recorded | Behaviour counselling in-person group sessions 1x week. Physical training group sessions 3x week | 11 wks | No | MET-hrs/week | Non | High |
| Brenner  (2019) (27) | Canada  (RCT) | N = 45 | 58.6  (SD ±9) | White  (80%) | ≥secondary school (82.2%) | Behaviour counselling by phone or email, every 3 weeks. Activity tracker data used to track progress.  Home based. | 12 wks  24 wks follow up | No | MVPA min/day | Very @12wks  Quite @24wks | Some concerns |
| Lynch  (2019)(28) | Australia  (RCT) | N = 83 | 61.6  (SD ±6.4) | Not recorded | University (45.8%) | Behaviour counselling in-person & by phone. 1x in-person. 5x calls  Workbook, activity monitor. Home based. Review of PA and goal setting. | 12 wks | No | MVPA min/week | Very | Some concerns |

Notes: Biobehavioral = Biobehavioral models of fatigue (29), MET = metabolic equivalent, MPA = moderate physical activity, MVPA = moderate vigorous physical activity, PA = physical activity, RCT = randomised controlled trial, RoB = Risk of bias assessment, SCT = Social cognitive theory (30), SEM = Socio ecological model (31), TPB = Theory of planned behaviour (32), TTM = Transtheoretical Model (33).
